# Combining pleasant Olfactory and BRAin stimulations in treatment-resistant depression (COBRA): study protocol for a randomized controlled trial

**DOI:** 10.3389/fpsyg.2024.1451096

**Published:** 2024-12-17

**Authors:** Laetitia Imbert, Cécilia Neige, Maylis Dumas, Moustafa Bensafi, Nathalie Mandairon, Jérôme Brunelin

**Affiliations:** ^1^Université Claude Bernard Lyon 1, Centre National de la Recherche Scientifique, Institut National de la Santé et de la Recherche Médicale, Centre de Recherche en Neurosciences de Lyon U1028 UMR5292, PSYR2, Bron, France; ^2^Le Vinatier, Psychiatrie Universitaire Lyon Métropole, Bron, France; ^3^Université Claude Bernard Lyon 1, Centre National de la Recherche Scientifique, Institut National de la Santé et de la Recherche Médicale, Centre de Recherche en Neurosciences de Lyon U1028 UMR5292, NEUROPOP, Bron, France

**Keywords:** repetitive transcranial magnetic stimulation (rTMS), major depressive disorder (MDD), olfaction, dorsolateral prefrontal cortex (DLPFC), reward system

## Abstract

**Background:**

Anhedonia, including social, physical, and less-known, olfactory, stands as a core symptom of major depressive disorder (MDD). At the neurobiological level, anhedonia has been associated with abnormal activity within the reward system, suggesting a key role for dopamine. Repetitive Transcranial Magnetic Stimulation (rTMS) has emerged as an innovative treatment for alleviating depressive symptoms. Stimulation of the dorsolateral prefrontal cortex (DLPFC) has been shown to both improve anhedonia and induce dopamine release. Moreover, research suggests that the efficacy of rTMS is improved when applied to an activated brain network rather than at rest. Our goal is to induce a dual activation of the reward system using a combined rTMS protocol and an intervention based on pleasant odorant exposure known to stimulate this system.

**Methods:**

In this randomized controlled trial, we propose to combine rTMS targeting the left DLPFC with pleasant odorant stimulation to alleviate depressive symptoms. A total of 80 patients with treatment-resistant MDD will be randomly assigned to two groups and will receive 50 sessions of either: 1- rTMS and hedonic olfactory stimulations, or 2- rTMS alone. We will conduct pre- and post-assessments measuring depression severity, physical, social, and olfactory anhedonia, as well as the connectivity and activity of brain regions involved in the pathophysiology of depression and the reward circuitry.

**Discussion:**

This study may strengthen the development of more effective rTMS interventions and pave the way for the establishment of rTMS combined with olfactory training as a safe, effective, and easily accessible treatment for MDD patients. In addition, this study will contribute to a better understanding of the mechanisms and physiopathology of MDD.

**Trial registration number:**

#NCT05661383.

## 1 Introduction

### 1.1 Background and rationale

#### 1.1.1 Major depressive disorder and anhedonia

Major depressive disorder (MDD) stands as the most common psychiatric disorder, affecting 4.4% of the world's population (World Health Organization, 2017). Among the clinical manifestations, anhedonia, the inability to experience pleasure, is one of the core symptoms of depression, affecting 70% of patients with MDD and linked to increased suicide risk. Furthermore, it is typically resistant to the first-line treatment (Kelly et al., 2022), making it one of the priorities of mental health research (Ducasse et al., 2021). This symptom appears to be rooted in a dysfunction of the reward system, i.e., hyperactivity in the prefrontal cortex (PFC) and hypoactivity in the ventral striatum (Gorwood, 2008), in which dopamine (DA) plays a key role. Furthermore, the symptom of motivational anhedonia has been linked to an abnormal availability of DA receptors in the ventral striatum (Peciña et al., 2017). Importantly, anhedonia can be physical and social, but it can also affect the senses, including olfaction.

Olfactory anhedonia has been shown to contribute to disrupted eating, social interactions, and mood (Clepce et al., 2010). Interestingly, the reward system, impaired in anhedonia, is closely connected to the olfactory system through the olfactory tubercle located in the ventral striatum, directly linked to the ventral tegmental area (Ikemoto, 2007). Olfactory stimulation has even been shown to activate DA neurons within the reward circuits in mice (Midroit et al., 2021) and brain regions of the mesocorticolimbic pathway in humans (Rolls et al., 2003).

Furthermore, several studies have shown that odors can improve mood and feeling of calm (Canbeyli, 2022), and that improved olfactory capabilities are associated with a reduction in depressive symptoms (Sabiniewicz et al., 2022) in participants without psychiatric disorders. While these findings are encouraging, the impact of such olfactory interventions in patients with MDD remains unclear.

#### 1.1.2 Repetitive transcranial magnetic stimulation protocol in MDD

Over the past decades, repetitive transcranial magnetic stimulation (rTMS) has emerged as an effective treatment for treatment-resistant symptoms in patients with MDD. This non-invasive brain stimulation technique modulates the activity and connectivity of a targeted brain network by applying a magnetic coil over the scalp. The effect depends on the stimulation parameters and the underlying brain network activity. It can result in either an increase or a decrease in the activity of the targeted network. Based on neuroimaging studies showing an imbalance in activity between the right and left dorsolateral prefrontal cortex (DLPFC) in patients with MDD (George et al., 1994; Grimm et al., 2008), it has been proposed to use either high-frequency rTMS over the left DLPFC or low-frequency rTMS over the right DLPFC to alleviate depressive symptoms. Although recent evidence-based guidelines report beneficial effects of rTMS on depressive symptoms (Lefaucheur et al., 2020), only 30% of patients achieved remission (Vida et al., 2023), leaving much room for optimization of rTMS protocols.

#### 1.1.3 Optimize rTMS protocol

A better knowledge of the underlying mechanisms of rTMS should help to optimize the clinical application of rTMS in depression. Several neuroimaging studies have shown that stimulating the left DLPFC leads to DA release in the ventral striatum in healthy participants (Strafella et al., 2001; Fonteneau et al., 2018), as well as in patients with MDD (Pogarell et al., 2006, 2007), and schizophrenia (Brunelin et al., 2011). These findings suggest that modulation of subcortical DA transmission may be a key mechanism underlying the therapeutic effects of rTMS.

Furthermore, the combination of rTMS with additional therapeutic strategies, including psychotherapy (Donse et al., 2018), cognitive behavioral therapy (Vedeniapin et al., 2010), and pharmacological treatments (Liu et al., 2014; but see Brunelin et al., 2014 for contradictory results), has been demonstrated to enhance the clinical efficacy of rTMS in depression. The same synergistic effect has also been observed with exposure therapy in individuals with tobacco use disorder (Dinur-Klein et al., 2014) and in patients with post-traumatic stress disorder (Isserles et al., 2013). These synergistic effects are thought to be due to the initial state of the neural network activation, that influences the effects of non-invasive brain stimulation (NIBS), with stimulation of an already activated network being more effective and enhancing the effects of the stimulation (Silvanto and Pascual-Leone, 2008).

Altogether, one may hypothesize that the dual-activation of the DA reward system with both TMS over the DLPFC and olfactory-based intervention with pleasant odorant inducing DA release may be particularly relevant in the treatment of anhedonia (Neige et al., 2024).

#### 1.1.4 Aim and hypotheses

This study aims to electrochemically stimulate the DA reward circuitry to alleviate anhedonia and achieve remission in patients with treatment-resistant MDD. We therefore propose to increase DA release from ventral tegmental area neurons and thereby increase ventral striatal activity, which is reduced in anhedonia. For this purpose, we will combine rTMS applied over the left DLPFC with hedonic olfactory stimulation (i.e., odorants with pleasant hedonic value), both known to modulate the DA reward system.

- By stimulating the DLPFC with rTMS, we aim to enhance its feedback control over subcortical structures.
- By using odorants with positive hedonic value known to recruit/activate the reward circuit, we aim to enhance DA release and thereby increase ventral striatal activity which is reduced in anhedonia,

Based on evidence linking anhedonia to reduced DA transmission and blunted mesocorticolimbic network functional connectivity (Phillips et al., 2023), we hypothesize that rTMS would alleviate depressive symptoms through changes in functional and effective connectivity within DA-related brain regions.

We propose that the combined approach may be more effective in enhancing DA release, as both methods stimulate DA activity, resulting in greater improvements in anhedonia and a more pronounced reduction in depressive symptoms compared to rTMS alone.

### 1.2 Objectives

#### 1.2.1 Primary objective

The main objective is to investigate the acute clinical effect of the combination of rTMS and hedonic olfactory stimulation on depressive symptoms compared to rTMS alone, measured as the number of patients who achieved remission after the treatment (number of patients with a MADRS_10_ < 10).

#### 1.2.2 Secondary objectives

##### 1.2.2.1 Clinical outcomes

We will assess the acute and long-term effects of the treatment on several clinical features of MDD: remission (MADRS_10_ < 10) and response (reduction of at least 50% in MADRS_10_ score), severity of self-rated depression (BDI_13_), physical and social anhedonia and apathy immediately after the treatment, after 1-month and 3-month follow-up.

##### 1.2.2.2 Behavioral outcome

We will investigate the effect of the treatment on cognitive biases using a free viewing task (see 4.2.2.1 Eye tracking free-viewing task), at baseline, after 1, 5, and the 50 sessions of rTMS.

##### 1.2.2.3 Brain connectivity and activity assessments

We will explore the effects of the treatment on brain connectivity and activity within the brain regions involved in the reward and olfactory processes. Additionally, we will also investigate the relationship between brain changes and clinical features changes.

##### 1.2.2.4 Olfactory anhedonia

Finally, our last objective will be to better characterize olfactory anhedonia (i.e., the impaired olfactory perception of pleasant odorants) by investigating its association with physical and social anhedonia, but also with early trauma. Olfactory anhedonia will be assessed before, immediately after treatment, after 1-month and 3-month follow-up through specific testing.

### 1.3 Trial design

This is a prospective, randomized, double-blind, and parallel-group controlled trial. The study aims to compare the clinical benefits of two interventions: rTMS alone and rTMS combined with olfactory stimulation.

## 2 Methods: participants, intervention, and outcomes

### 2.1 Study setting

This trial is a single-center study. All the patients will be recruited at Centre Hospitalier Le Vinatier (Lyon, France). Healthy volunteers, matched for sex and age, will also be recruited to compare several clinical and biological features at baseline. All measurements and interventions will be performed at the same site, to ensure a uniform and controlled study environment.

### 2.2 Eligibility criteria

#### 2.2.1 Patients with MDD

To be eligible for the study, patients must meet the following criteria: be 18 years or older, have a primary diagnosis of single or recurrent non-psychotic major depressive disorder (unipolar or bipolar) episode according to the Diagnostic and Statistical Manual of Mental Disorders, fifth edition (DSM-5) criteria, scored 20 or more on the 10-item Montgomery and Asberg Depressive Rating Scale (MADRS) (Montgomery and Åsberg, 1979) and scored higher than 2 on the MADRS anhedonia score (item #8). All patients should be able to speak and read French, and sign a written informed consent.

Patients with unipolar depression should be on stable antidepressant medication for at least 4 weeks prior to inclusion with no mood changes (after at least one previous treatment failure). Patients recruited for this study had a treatment-resistant depressive episode, defined as failure to respond to at least two antidepressant regimens despite adequate dosage, duration and adherence to treatment (McIntyre et al., 2023). Patients will remain on the same stable antidepressant medication throughout the trial without any changes. Benzodiazepines are not allowed.

Patients with bipolar depression should have treatment-resistant bipolar depression at enrolment, despite optimized mood-stabilizing treatment of adequate duration and dosage (for at least one month). Throughout the study period, patients will be on stable mono- or bi- therapy with mood stabilizers, without antidepressants or benzodiazepines (Bulteau et al., 2019).

Augmentation strategy with antipsychotics is allowed.

Participants will be not eligible if they have: anosmia [as verified by the European Test of Olfactory Capabilities (ETOC) (Thomas-Danguin et al., 2003)], whether congenital, or due to upper respiratory tract infection, nasal and/or sinus disease, brain injury or nasal surgery; neurological disorders; concurrent comorbid psychiatric disorders or substance abuse (except tobacco); contraindications to TMS (implanted medical devices or metallic foreign body in the head); contraindications to MRI; history of non-response to treatments based on sensory stimulation or rTMS; pregnant or breastfeeding mothers (controlled by urine pregnancy tests); a conservatorship or guardianship order.

Patients will be free to withdraw from the study without having to justify their decision. The principal investigator may end the patient's participation due to loss to follow-up, severe intercurrent pathology, modification/discontinuation of treatment, or change of diagnosis.

#### 2.2.2 Healthy volunteers

A total of 40 healthy volunteers will be recruited. They must meet the following criteria: be aged 18 years or older, be able to speak and read French and sign a written informed consent before intervention procedure. They will be not included if they have: current or a history of psychiatric disorder (based on a structured interview with a trained psychiatrist), anosmia (as verified by the ETOC), a current pharmacological treatment, or contraindications to TMS.

### 2.3 Intervention

All stimulations for therapeutic intervention will be delivered using a MagPro X100 (MagVenture, Mag2Health, France) equipped with a 65 mm figure-of-eight coil with active cooling (MCF-Cool B65). Patients will be comfortably installed in a dedicated room for brain stimulation. During each session, the 3D T1-weighted images of all participants will be used for neuronavigation and the position and orientation of the coil will be monitored using a neuronavigation system (Syneika system, France).

At the first session, the primary motor cortex (M1) hand area will be located by identifying the optimal scalp sit (hotspot) that elicits the largest amplitude of motor evoked potentials (MEPs) in the right hand's first dorsal interosseus (FDI) muscle for a given intensity. The resting motor threshold (rMT) will then be established as the lowest stimulation intensity to evoke at least 5 MEP of 50 μV peak-to-peak amplitude from 10 consecutive (Rossini et al., 1994).

For the rTMS protocol, the coil will be positioned over the left DLPFC based on the individual anatomical T1-weighted MRI. The targeted left DLPFC will be identified based on a predefined identification by the Syneika neuronavigation system, i.e., the junction between areas BA9 and BA46, according to the algorithm proposed by Mylius et al. (2013). The exact same target will be maintained for each rTMS session to ensure precise and reproducible placement of the stimulation coil.

The rTMS protocol includes 50 sessions over 10 days (i.e., 5 sessions per working day, 1 h apart, for 2 weeks). Each session lasts 9min40 and involves 1,800 pulses at 90% rMT, delivered in bursts of 3 pulses at 50 Hz, repeated at 200 ms intervals for 2 s (i.e., at 5 Hz). This particular rTMS protocol is called intermittent theta-burst stimulation (iTBS). A 2-second train of iTBS is repeated every 10 s (Cole et al., 2022).

In the iTBS combined with olfactory stimulation group, pleasant odorants will be delivered through passive diffusers placed in the room dedicated to the iTBS protocol, during the 9min40 treatment. During the inclusion phase, from the 20 odorants presented in a hedonic olfactory test, 10 are considered as potentially diffusible during the treatment for two reasons: (1) they are known to be pleasant, (2) we have tested them so that they diffuse well and retain their pleasant odorant properties. Of these 10, 5 are single molecules known to be pleasant (Chalençon et al., 2022; Sezille et al., 2014) and 5 are blended odorants that are appreciated as much as single molecule odorants (Chen et al., 2022). The 3 highest rated odorants will be selected, and one will be randomly chosen and diffused at 20 Pa during each iTBS session using a heat diffusion system.

### 2.4 Outcomes

#### 2.4.1 Primary outcome

The primary outcome is the number of patients meeting remission criteria within each post-treatment group. Remission is defined as a MADRS score of 10 or less (Zimmerman et al., 2004) on a scale of 0–60, assessed at the end of 50 iTBS sessions.

#### 2.4.2 Other clinical and behavioral outcomes

Several clinical and behavioral features will be assessed using:

- the 10-item MADRS total scores to assess depression severity.
- the number of responders, defined as those who show a reduction of at least 50% in their MADRS score.
- the 13-item Beck Depression Inventory (BDI) (Gould, 1982), to assess self-rated depression.
- the 11-item Young Mania Rating Scale (YMRS) (Young et al., 1978), to assess manic states.
- the Childhood Trauma Questionnaire (CTQ) (Bernstein et al., 2003) to study early life adversity.
- the Chapman Social Anhedonia Scale and the Chapman Physical Anhedonia Scale (Chapman et al., 1976) to investigate respectively physical and social anhedonia.
- the Snaith-Hamilton Pleasure Scale (SHAPS) (Snaith et al., 1995) to assess global anhedonia.
- the Apathy Evaluation Scale (AES) (Marin et al., 1991) to assess apathy.
- the Free-Viewing task (FVT) to assess cognitive biases, more precisely, early and sustained emotional attentional biases.

These outcomes will be assessed before and after the 50 iTBS sessions. The same outcomes will also be assessed at 1 and 3 months after treatment and compared between the two groups. In addition, the relationship between early life adversity and depression severity will be examined, as well as its association with the evolution of depressive symptoms and especially anhedonia.

#### 2.4.3 Neurobiological outcomes

The brain connectivity and activity within the brain regions involved in the reward and olfactory circuitry we will be explored through functional Magnetic Resonance Imaging (fMRI) and dual-site transcranial magnetic stimulation protocol. This will be assessed before and after the treatment, and compared between the two groups. The relationship between changes in neurobiological outcomes and changes in clinical features will be also investigated.

#### 2.4.4 Olfactory anhedonia

Olfactory anhedonia will be assessed using an olfactory test in which the hedonic value of odorants will be evaluated before and after the treatment, as well as at 1 and 3 months after the treatment, and compared between the two groups. The relationship between olfactory anhedonia and physical and social anhedonia will be investigated, as well as its association with early life adversity.

### 2.5 Participants timeline

Table 1 provides an overview of the schedule of enrolment, interventions, and assessments.

**Table 1:** Schedule of the study.

	**Study period**						
	**Patients with MDD**						**Healthy volunteers**
	**Inclusion**	**Allocation**	**Post-Allocation**		**Follow-up**		**Experimentation**
**Timepoint (week)**	**-1**	**0**	**1**	**2**	**6**	**14**	**1**
**Inclusion:**							
Eligibility screen	**X**						**X**
Informed consent	**X**						**X**
Demographics	**X**						**X**
Allocation		**X**					**X**
**Interventions:**							
iTBS alone			↔				
iTBS combine with odorants			↔				
**Assessments:**							
MADRS	**X**	**X**		**X**	**X**	**X**	
BDI		**X**		**X**	**X**	**X**	**X**
CTQ		**X**					**X**
Chapman scales		**X**		**X**	**X**	**X**	
SHAPS		**X**		**X**	**X**	**X**	**X**
AES		**X**		**X**			**X**
ETOC/hedonic perception		**X**					**X**
FVT		**X**		**X**			
MRI		**X**		**X**			
Dual-site TMS		**X**		**X**			**X**

iTBS, intermittent Theta Burst Stimulation; MDD, Major Depressive Disorder; MADRS, Montgomery and Asberg Depressive Rating Scale; BDI, Beck Depression Inventory; CTQ, Childhood Trauma Questionnaire; SHAPS, Snaith-Hamilton Pleasure Scale; AES, Apathy Evaluation Scale; ETOC, European Test of Olfactory Capabilities; FVT, Free-Viewing Task; MRI, Magnetic Resonance Imagery; TMS, Transcranial Magnetic Stimulation.

### 2.6 Sample size

#### 2.6.1 Patients with MDD

The sample size was estimated using EasyMed online software (https://easymedstat.fr), with an a priori power analysis based on a comparison of two binomial proportions (approximation of arscin).

We expect an approximately twofold increase in the remission rate for the iTBS combined with olfactory training group, compared to the iTBS alone group. This expectation is based on results from similar protocols using dual activation of the same neural network, such as the combination of high-frequency rTMS and visual smoking cues in individuals with tobacco use disorder, which reported 1.8-fold higher remission rate in the combination group than in the rTMS group alone (Dinur-Klein et al., 2014), and deep TMS with exposure in patients with post-traumatic stress disorder (Isserles et al., 2013), which reported a 3.5-fold higher response rate in the combination group than in the rTMS alone group.

Based on previous studies investigating the clinical efficacy of rTMS in treatment-resistant depression in both RCTs (Vida et al., 2023) and naturalistic settings (Bouaziz et al., 2023), we predict an approximate remission rate of 30% for the iTBS alone group. Thus, the expected remission rate for the iTBS combined with olfactory training group is estimated to be 60%. With a chi-squared test, an α risk of 5%, and a ß-power of 80%, a total of 74 MDD patients (37 per group) are required to be included.

As all patients enrolled in this trial will receive active treatment, we anticipate a low attrition rate (7%) and therefore plan to recruit a total of 80 patients with MDD (40 per group).

#### 2.6.2 Healthy volunteers

In order to provide evidence of impaired effective connectivity during odors perception in patients with major depressive disorder (MDD) compared to participants without a psychiatric disorder, a total of 40 healthy volunteers will be included in the study.

The final sample of the study will consist of 120 participants: 40 patients with MDD in the iTBS alone group, 40 patients with MDD in the iTBS combined with olfactory training group and 40 healthy volunteers.

### 2.7 Recruitment

#### 2.7.1 Patients with MDD

Patients will be recruited from specialized units for treatment-resistant depression and bipolar disorder at Le Vinatier, psychiatric hospital (Regional Expert centers). These units provide consultation to a large number of patients, from which the patients will be recruited. In addition, several recruitment strategies will be used, including flyers/brochures/posters, emails and websites/social media pages dedicated to patient recruitment in relation with the URPS Laboratoire participatif, AURA region.

#### 2.7.2 Healthy volunteers

To recruit the healthy controls, several calls for participation will be relayed by: posters in public places, publications on social network and websites, emails send to specific diffusion lists.

## 3 Methods: assignment of intervention

### 3.1 Sequence generation

Patients will be randomly assigned (1:1) to receive either iTBS alone or iTBS combined with pleasant olfactory stimulation. Computer-generated random numbers using the Sealed Envelope platform (https://www.sealedenvelope.com) will be used to generate the allocation sequence. Block of 2, 4, and 6 will be used to stratified the randomization.

### 3.2 Allocation concealment mechanism

The allocation sequence will be handled by an independent person from the Administrative Department of the Hospital to ensure that it is not disclosed to those responsible for enrolling and allocating patients.

### 3.3 Implementation

All the included participants will be assigned to a unique anonymous identification code, consisting of the group assignment number, the inclusion number and the patient's initials (first letter of last name and first letter of the first name). The person providing care will then administer the appropriate treatment associated with the inclusion number, according to the order of assignment.

### 3.4 Blinding

To ensure that patients are blinded to the allocated treatment, the information notice distributed to patients will state that odorants will be diffused during the TMS sessions, but at either a supraliminal or subliminal concentration, meaning that it may be normal not to smell anything. In addition, both the clinicians who complete the clinical scales and the researchers who analyze the data will be blinded to the allocation.

## 4 Methods: data collection, management, and analysis

### 4.1 Data collection methods

#### 4.1.1 Plans for assessment and collection of outcomes

The clinician-rated questionnaires will be scored by experienced psychiatrists who have undergone training to ensure homogeneity of scoring across raters. It is noteworthy that these scales are commonly used in research in the field of depression, and that all the psychiatrist-raters are experts in the treatment of depression.

### 4.2 Description of clinical scales and assessment tools

#### 4.2.1 Clinical assessments

##### 4.2.1.1 Symptoms severity

The MADRS is a clinician-rated questionnaire designed to assess the depression severity through 10 items. Each item has six levels of severity. Depression severity is also measured by a self-rated questionnaire, the 13-item BDI, in which participants will rate each item on a severity scale from 0 to 3. Manic states severity will be assessed with the 11-item YMRS. The total score can range from 0 to 60 (with 7 items rated 0 to 4, and 4 items rated 0 to 8).

##### 4.2.1.2 Early life adversity

Childhood trauma and maltreatment negatively affect rTMS-left DLPFC treatment outcome in patients with MDD (Hu et al., 2021). Therefore, the short form of the CTQ will be used, which includes 28 items, rated from 0 to 5 according to the occurrence of the event experienced. It allows to identify 5 different types of trauma: emotional neglect, physical neglect, emotional abuse, physical abuse, and sexual abuse. Subtotals can be calculated for each type of trauma to provide a nuanced understanding of the participant's childhood trauma experiences.

##### 4.2.1.3 Anhedonia

Physical and social anhedonia will be assessed using the Chapman Social Anhedonia Scale (SAS) and the Chapman Physical Anhedonia Scale (PAS). The SAS is a 40-item dichotomously scored self-report questionnaire measuring social anhedonia which reflects a preference for solitary activities. Scores can range from 0 to 40, with higher scores indicating a reduced ability to experience pleasure from social and interpersonal experiences. The PAS is a 61-item dichotomously scored self-report questionnaire that measures physical anhedonia which refers to the inability to experience pleasures from stimuli such as food, touch, smell, sex, temperature, movement, sound and physical sensations. Scores range from 0 to 61, with higher scores indicating a reduced pleasure from physical stimuli.

The second assessment tool is the SHAPS, a 14-item self-report questionnaire. Participants rate their agreement with statements using “strongly disagree,” “disagree,” “agree,” or “strongly agree.” Scores range from 0 to 14, with higher scores indicating greater levels of anhedonia.

##### 4.2.1.4 Apathy

The Apathy Evaluation Scale (AES) has been developed to quantify and characterize apathy in adults. The scale consists of 18 items assessing the emotional, cognitive and behavioral aspects of apathy in the month prior to the assessment. The rating is based on a 4-point Likert scale, with 1 corresponding to “not at all true” and 4 corresponding to “very true.” The version used in this study is the one completed by the participants themselves (AES-S).

##### 4.2.1.5 Olfactory capabilities

The European Test of Olfactory Capabilities (ETOC) will be used to assess the ability to identify and detect odorants. This test involves the presentation of 16 odorants at supraliminal concentrations. In each trial, 4 tubes are presented to the participants, only one of which contains the odorant. The aim of the task is to identify the tube containing the odorant, followed by identifying the specific odorant from a set of 4 propositions. Both tests are scored out of 16.

##### 4.2.1.6 Olfactory anhedonia

In addition, an olfactory hedonic judgment test will be assessed. It consists of the presentation of 20 odorants (with different hedonic value, from pleasant to unpleasant), 15 of which are monomolecular at a concentration of 20 Pa: isoamyl acetate, 2-phenylethanol, benzaldehyde, L-carvone, linalool, trans-anethol, benzyl-acetate, eucalyptol, cis-3-hexanol, citronellol, D-limonene, eugenol, n-caproic acid, trimethylamine, butyric acid, thioglycolic acid, and 5 are blended odorants: lemon/sweet orange/rosemary/bergamot/bitter orange/tree leaf/lavender/clary sage, linalool/d-limonene/linalyl acetate/beta-caryophyllene/beta-pinene/citral/geraniol/alphapinene/ eucalyptol/l-beta-bisabolene/menthone/geranyl acetate/2,6-dimethyl-5-heptenal/terpinolene, sweet orange/lavender/rosemary/bergamot/basil, vanilla extract/orange/ylang-ylang, and ylang-ylang/palmarosa/sweet orange/isoeugenol.

Participants will be allowed to freely smell each odorant presented in a randomized order. Then, participants will be asked to rate each odorant in terms of liking (“Is the smell pleasant?”), wanting (“Do you want to smell it again?”), intensity (“Is the smell intense?”), familiarity (“Is the smell familiar?”) (Chalençon et al., 2022). Each odorant will be rated on an analog scale ranging from 1 to 9 (1 for “Not at all,” 5 for “Neutral,” and 9 for “Extremely”).

#### 4.2.2 Behavioral assessment

##### 4.2.2.1 Eye tracking free-viewing task

The Free Viewing Task (FVT) will assess attentional emotional bias before the treatment and after 1, 5, and 50 rTMS sessions, as it correlates with depression severity (Imbert et al., 2024). This makes it a relevant outcome to evaluate before and after treatment, providing objective measures of improvement. In the task, pairs of faces: one emotional (happy or sad) and one neutral, are presented randomly for 3,500 ms to the right and left of a fixation cross. The inter-stimulus interval is randomized among 1,250, 1,500, or 1,750 ms, and the next pair appears only when the participant fixates on the cross. A total of 32 pairs (16 happy- neutral, 16 sad-neutral pairs), with 16 different identities (8 males, 8 females), will be presented. The faces are extracted from the FACES database (Ebner et al., 2010).

Throughout the task, gaze position and eye movements are recorded using an eye-tracking system (SMI SensoMotoric Instruments with BeGaze 3.6.52, Teltow, Germany).

We will assess early and sustained attention allocation using eye-movement recordings. Sustained attention will be measured by dwell time (DT), the total time spent fixating on faces. The emotional bias score will be the difference between DTs for emotional and neutral faces. Early attention will be measured by the orientation of the first saccade, the first face the participant looks at. The laterality quotient will be the difference between the number of first saccades to emotional vs. neutral faces, divided by the total trials. Positive values indicate a bias toward emotional faces; negative values indicate a bias toward neutral faces.

#### 4.2.3 Neurobiological investigations

##### 4.2.3.1 Functional connectivity with magnetic resonance imaging

The MRI sequences will include, for anatomical measurements, a sagittal 3D T1-weighted sequence that will be acquired in about 8 min. The standardized parameters will ensure good contrast and whole-brain coverage with 0.8-mm isotropic resolution. A fieldmap will be acquired to correct for geometrical distortions induced by the EPI sequence. For functional measurements through changes in BOLD T2^*^-weighted signals, a 2D axial gradient echo EPI sequence will be acquired in 12 min. The standardized parameters will ensure maximum brain coverage with 2.7 mm isotropic voxels, a repetition time equal to 1.7 s, and 420 repetitions for ensuring statistical significance could be reached during the following analyses. Participants will be instructed to keep their eyes closed during the functional acquisition.

All MRI acquisitions will be performed at the CERMEP Multimodal Imaging Platform (Lyon, France) using a 3-Tesla MR Siemens Prisma scanner with a 64-channel head coil.

Imaging data will be pre-processed and analyzed according to the latest standard procedures available at the time of analysis.

##### 4.2.3.2 Effective connectivity with Dual-site transcranial magnetic stimulation

The MagStim 200^2^ BiStim^2^ dual-site TMS (MagStim, United Kingdom), connected to two 40 mm figure-of-eight coils, will be used to probe effective connectivity from the left DLPFC to the left M1 both at rest and during odor perception (pleasant and unpleasant odorants) sent using an olfactometer (Wang et al., 2020; Neige et al., 2023). When the olfactometer (Sezille et al., 2013) detects the exhalation phase, it initiates an airflow through tubes containing the odorant for 6 seconds. This odorant-charged airflow is delivered to the participant via nasal canula. At 600 ms following the onset of the inhalation phase, which represents the maximum conscious representation of the odorant (Kato et al., 2022), the olfactometer trigs the TMS to deliver a pulse to the DLPFC. After a brief delay, a trigger is sent to the TMS connected to M1 to administer a pulse.

This measure will be realized before and after the 50 sessions of iTBS. Motor-evoked potentials (MEP) will be recorded using surface electromyography (BIOPAC, United States of America) from the right FDI. The conditioned MEP peak-to-peak amplitude evoked by dual-site TMS will be compared to the MEP amplitude evoked by TMS applied over M1 alone (see Neige et al., 2023) for the detailed protocol of this experiment.

#### 4.2.4 Plans to promote participant retention and complete follow-up

##### 4.2.4.1 Patients with MDD

To encourage adherence to the protocol and to compensate for the time spent in the hospital, participants will receive 150 euros to cover travel, food expenses, and MRI sessions. The given amount will depend on the number of visits attended.

To minimize loss to follow-up, each participant will receive an individualized schedule of all visits at the beginning of their inclusion. Additionally, the research assistant will contact participants a few days before their scheduled appointments as a reminder.

##### 4.2.4.2 Healthy volunteers

A single visit is planned for healthy volunteers, thus limiting the loss rate. They will receive 50 euros for their participation in the study, with a further 10 euros to cover travel costs.

### 4.3 Data management

Sociodemographic and clinical data will be collected using a Case Report Form (CRF) that is stored in a secure cabinet. All the data will be reported in a secure tabular file stored on a secure server. Two-person double checks will be performed to verify the accuracy of the reported data.

MRI data will be accessible via the secure server of the neuroimaging platform and will be stored on the hospital's server.

### 4.4 Statistical methods

#### 4.4.1 Statistical methods for analyzing primary and secondary outcomes

Statistical analysis will be performed using R studio (Boston, USA, with the latest version at the time of the analysis) by an experimenter blinded to treatment allocation. The alpha threshold will be fixed at 0.05. Parametric tests will be performed only when the assumptions for their application are met, otherwise, non-parametric tests will be performed.

##### 4.4.1.1 Participants characteristics at inclusion

Baseline differences between the two groups in terms of socio-demographic factors (age, gender, educational level, laterality, smoking status) and clinical characteristics (depression severity, illness duration, episode duration, treatment, level of early life adversity) will be analyzed using independent samples *t-*tests and chi-squared tests or non parametric tests depending on the distribution of the data.

##### 4.4.1.2 Clinical data analysis

The proportion of remissions and responses will be investigated using chi-squared tests or Fisher's exact test. Changes in scores of depression severity, anhedonia and olfactory capabilities over the study period within the two groups will be analyzed using a mixed 2-way repeated measures ANOVA (GROUP^*^TIME) or a generalized linear mixed model depending on the distribution of the data. Pearson correlations will be performed to examine the relationship between olfactory anhedonia and pre-treatment physical and social anhedonia, as well as the relationship between childhood trauma severity and response to treatment, the relationship between childhood trauma severity and anhedonia (physical, social, and olfactory), and the relationship between childhood trauma severity and depression severity. Type of medication and type of depression (unipolar vs. bipolar) will be included in the analysis. We did not expect differences in response between patients with unipolar and bipolar depression.

##### 4.4.1.3 Behavioral data analysis

Changes in early and sustained attention within the two groups will be investigated using a mixed 2-way repeated measures ANOVA (GROUP^*^TIME) or a generalized linear mixed model, depending on the distribution of the data. Pearson correlations will be performed to examine the relationship between changes in depression severity and changes in emotional attentional biases.

##### 4.4.1.4 Neurobiological data analysis

The fMRI data will be preprocessed and analyzed according to the most recent standard procedures in the field available at the time of analysis. For the dual-site TMS protocol, a ratio of the MEP amplitude evoked by the dual-site TMS to the MEP amplitude evoked by single TMS over M1 will be performed. This ratio will be analyzed using a mixed 2-way repeated measures ANOVA (GROUP^*^TIME) or a generalized linear mixed model depending on the distribution of the data. Pearson correlations or multiple linear regressions will be performed between neurobiological and clinical and behavioral data.

#### 4.4.2 Withdrawals and missing data

All participants will be included in an intention-to-treat (ITT) analysis, based on their initial group assignment, even if they withdraw or fail to complete the study. For withdrawn participants, their data will still be analyzed as if they completed the study, and missing data will be imputed using the Last Observation Carried Forward (LOCF) method. Only patients with a pre-treatment evaluation and at least one post-treatment evaluation will be included in the analysis.

## 5 Methods: monitoring

### 5.1 Data monitoring

#### 5.1.1 Composition of data monitoring committee

The study sponsor will oversee data monitoring and schedule several monitoring visits throughout the study: one after the first inclusion and one annually thereafter.

#### 5.1.2 Interim analysis

Given the pilot nature of this study, we plan to conduct an interim efficacy analysis when 40 patients (50% of the total sample) have been included. This analysis will allow for the earliest detection of the benefit of adding hedonic olfactory stimulation, thus accelerating its availability to patients. During this interim analysis, the same primary and secondary analyses will be performed as at the end of the study. To reduce a type I error (related to α-risk inflation), the spending function of α risk proposed by O'Brien and Fleming (1979) will be used, with a stopping boundary (expressed in *P*-value) set at 0.005 for the interim analysis and 0.048 for the final analysis (Schulz and Grimes, 2005). The study will be stopped if a statistically significant difference in remission rates between the two groups is observed immediately after treatment. No futility analysis is planned.

### 5.2 Harms

Safety assessment will be conducted in accordance with French law. Adverse events attributable to treatment will be verified, assessed and categorized by the principal investigator in the appropriate section of the CRF. This includes any adverse events observed by the investigator as well as any adverse events reported by the participant.

### 5.3 Auditing

By signing the protocol, the investigator agrees that the sponsor, its representative and regulatory agencies have direct access to its study records for the purpose of an audit or an inspection. These personnel, bound by professional secrecy, will not divulge any personal identity or any personal medical information. In all cases, the sponsor will assist the investigator in preparing for an inspection by a regulatory authority.

## 6 Ethics and dissemination

### 6.1 Research ethics approval

This trial involving human participants has been reviewed and approved by ethics committee on 14 November 2022 (CPP IDF VII 2022-A1967-36). The study and has been registered on ClinicalTrial.gov (trial registration number NCT05661383) before its completion on 12 December 2022.

### 6.2 Consent

The study will be conducted in accordance with the Declaration of Helsinki.

Before consent is obtained, each patient will receive an information notice explaining, in comprehensible terms, the purpose of the study, the course of the study, associated risks and benefits, as well as their right to refuse to participate in the study and to withdraw at any time. An inclusion visit will be then scheduled, during which the investigator will reiterate this information and answer any questions the participant may have. Only after ensuring complete understanding, written informed consent will be obtained from a psychiatrist involved in the study. Both parties will sign copies of the consent form, with one copy given to the participant and the other kept by the investigator.

### 6.3 Confidentiality

All participant-related information will be collected in a paper CRF and reported in an electronic database. The data flow has been declared and approved by the CNIL (National Data Privacy Commission). To ensure confidentiality, all participant documents will be anonymized and will display only an ID code consisting of the treatment group number, serial number and patient initials.

### 6.4 Declaration of interest

The authors declare that the research was conducted in the absence of any commercial or financial relationship that could be construed as a potential conflict of interest.

### 6.5 Access to data

The study sponsor will have full access to the final data set at the end of the trial. Anonymized data will be made available to those responsible for statistical analysis and may be shared with other researchers upon reasonable request and in accordance with EU regulation - GDPR.

### 6.6 Ancillary and post-trial care

Patients will remain under double-blind throughout the follow-up period with no change in treatment. Follow-up will be carried out for a period of 3 months. After inclusion, patients may not participate in any other clinical research will be permitted during the study.

## 7 Dissemination policy

### 7.1 Communication of trial results

Results will be presented at national and international scientific meetings and conferences. Articles will be submitted to international peer-reviewed journals. Anonymized dataset may be shared with other scientists upon reasonable request. The sponsor of the study and the funders play no role in the design of the study, no role during its execution, analyses, interpretation of the data, or decision to publish the results.

### 7.2 Authorship

JB and NM designed the study and lead the protocol development. JB was responsible for the global methodology, NM and MB for the olfactory methodology and CN for the dual-site TMS methodology. CN, LI and MD will perform the experiments and statistical analysis. LI and CN wrote the first draft of the manuscript and all authors contributed to and approved the final version of the manuscript.

### 7.3 Plans to give access to the full protocol, participant level-data and statistical code

This manuscript is the full protocol of the study. Anonymized dataset and statistical code may be shared with other scientists upon reasonable request.

## 8 Discussion

Thirty percent of individuals suffering from MDD are resistant to pharmacological treatments, underscoring the urgent need for novel therapeutic alternatives to treat depression. rTMS has been proposed as an innovative tool to alleviate depressive symptoms. Although approved by health authorities in certain countries for the treatment of major depression, the considerable heterogeneity in clinical response leaves much room for optimizing its application in clinical settings. Here, we propose the first randomized, double-blind, controlled trial to investigate clinical and biological effects of the combination of hedonic olfactory and brain stimulations.

The iTBS protocol proposed in this study seems to be appropriate, considering that other studies have already shown its efficacy on depressive symptoms (Duprat et al., 2016; Cole et al., 2022; Wilkening et al., 2022), its safety across numerous psychiatric condition (Caulfield et al., 2022; Brunelin et al., 2022) and that accelerated protocols allows a better access to treatment for patients (Mondino et al., 2021). The use of pleasant odorants to modulate neural activity within the reward system is grounded in the close connection between this system and the olfactory one, through structures like the olfactory tubercle. Moreover, stimulation of the left DLPFC is thought to result in the release of subcortical DA (Fonteneau et al., 2018; Brunelin et al., 2011; Strafella et al., 2001), which may help stimulate the reward circuitry.

The knowledge gained from in this project will strengthen the development of more effective NIBS strategy and pave the way for the establishment of NIBS combined with olfactory training as a safe, effective, and easily accessible treatment for patients with both unipolar and bipolar major depressive episode.

In addition, anhedonia can also be considered as a transdiagnostic symptom that is common to psychiatric disorders, including schizophrenia and post-traumatic stress disorder. From this perspective, a similar protocol could potentially be applied to address this symptom across a range of psychiatric conditions.

Finally, this study will provide a better understanding of the mechanisms and pathophysiology of major depressive episodes and depressive symptoms (especially anhedonia), as well as the mechanisms of action of rTMS. Dual-coil TMS, iTBS protocol and fMRI will allow us to better understand the role of the DLPFC in anhedonia.

## 9 Limitation

Ensuring that participants are blind to their assigned group is a major limitation. To limit this bias, participants are informed that the odorants may be at a supraliminal or infraliminal concentration, which could explain the difference in perception. This approach ensures that the participant believes in the presence of an odorant. To maintain the evaluators' blindness, participants are asked not to share their feelings about the treatment.

Finally, we are constantly in an environment rich in sensory stimuli, and the design of this study does not allow us to control for patients' previous exposure to odorants or other forms of sensory stimuli. However, the verification of the absence of anosmia, the fixed list of odorants presented, the selection of the three most appreciated odorants, and the time-limited exposure to odorants during the iTBS sessions provide a clear methodological control for this study.

## References

[B1] Bernstein D. P. Stein J. A. Newcomb M. D. Walker E. Pogge D. Ahluvalia T. . (2003). Development and validation of a brief screening version of the Childhood Trauma Questionnaire. Child Abuse Negl. 27, 169–190. 10.1016/S0145-2134(02)00541-0 12615092

[B2] Bouaziz N. Laidi C. Bulteau S. Berjamin C. Thomas F. Moulier V. . (2023). Real world transcranial magnetic stimulation for major depression: a multisite, naturalistic, retrospective study. J. Affect. Disord. 326, 26–35. 10.1016/j.jad.2023.01.070 36708953

[B3] Brunelin J. Bouaziz N. Dollfus S. Kallel L. Jardri R. Rachid F. . (2022). Safety of “accelerated” rTMS protocols with twice-daily sessions in patients with schizophrenia–a comment on Caulfield et al. J. Psychiatr. Res. 156, 754–757. 10.1016/j.jpsychires.2022.08.025 36088124

[B4] Brunelin J. Jalenques I. Trojak B. Attal J. Szekely D. Gay A. . (2014). The efficacy and safety of low frequency repetitive transcranial magnetic stimulation for treatment-resistant depression: the results from a large multicenter French RCT. Brain Stimul. 7, 855–863. 10.1016/j.brs.2014.07.040 25192980

[B5] Brunelin J. Szekely D. Costes N. Mondino M. Bougerol T. Saoud M. (2011). Theta burst stimulation in the negative symptoms of schizophrenia and striatal dopamine release. An iTBS-[11C] raclopride PET case study. Schizophr. Res. 131, 264–265. 10.1016/j.schres.2011.05.019 21669513

[B6] Bulteau S. Beynel L. Marendaz C. Dall'Igna G. Peré M. Harquel S. . (2019). Twice-daily neuronavigated intermittent theta burst stimulation for bipolar depression: a randomized sham-controlled pilot study. Neurophysiol. Clin. 49, 371–375. 10.1016/j.neucli.2019.10.002 31761447

[B7] Canbeyli R. (2022). Sensory stimulation via the visual, auditory, olfactory and gustatory systems can modulate mood and depression. Eur. J. Neurosci. 55, 244–263. 10.1111/ejn.15507 34708453

[B8] Caulfield K. A. Fleischmann H. H. George M. S. McTeague L. M. (2022). A transdiagnostic review of safety, efficacy, and parameter space in accelerated transcranial magnetic stimulation. J. Psychiatr. Res. 152, 384–396. 10.1016/j.jpsychires.2022.06.038 35816982 PMC10029148

[B9] Chalençon L. Thevenet M. Noury N. Bensafi M. Mandairon N. (2022). Identification of new behavioral parameters to assess odorant hedonic value in humans: a naturalistic approach. J. Neurosci. Methods 366:109422. 10.1016/j.jneumeth.2021.109422 34826503

[B10] Chapman L. J. Chapman J. P. Raulin M. L. (1976). Scales for physical and social anhedonia. J. Abnorm. Psychol. 85:374. 10.1037/0021-843X.85.4.374 956504

[B11] Chen J. Zhang N. Pei S. Yao L. (2022). Odor perception of aromatherapy essential oils with different chemical types: influence of gender and two cultural characteristics. Front. Psychol. 13:998612. 10.3389/fpsyg.2022.998612 36438419 PMC9686375

[B12] Clepce M. Gossler A. Reich K. Kornhuber J. Thuerauf N. (2010). The relation between depression, anhedonia and olfactory hedonic estimates—a pilot study in major depression. Neurosci. Lett. 471, 139–143. 10.1016/j.neulet.2010.01.027 20097263

[B13] Cole E. J. Phillips A. L. Bentzley B. S. Stimpson K. H. Nejad R. Barmak F. . (2022). Stanford neuromodulation therapy (SNT): a double-blind randomized controlled trial. Am. J. Psychiatry 179, 132–141. 10.1176/appi.ajp.2021.20101429 34711062

[B14] Dinur-Klein L. Dannon P. Hadar A. Rosenberg O. Roth Y. Kotler M. . (2014). Smoking cessation induced by deep repetitive transcranial magnetic stimulation of the prefrontal and insular cortices: a prospective, randomized controlled trial. Biol. Psychiatry 76, 742–749. 10.1016/j.biopsych.2014.05.020 25038985

[B15] Donse L. Padberg F. Sack A. T. Rush A. J. Arns M. (2018). Simultaneous rTMS and psychotherapy in major depressive disorder: clinical outcomes and predictors from a large naturalistic study. Brain Stimul. 11, 337–345. 10.1016/j.brs.2017.11.004 29174304

[B16] Ducasse D. Dubois J. Jaussent I. Azorin J. M. Etain B. Gard S. . (2021). Association between anhedonia and suicidal events in patients with mood disorders: a 3-year prospective study. Depress. Anxiety 38, 17–27. 10.1002/da.23072 32652874

[B17] Duprat R. Desmyter S. van Heeringen K. Van den Abbeele D. Tandt H. Bakic J. . (2016). Accelerated intermittent theta burst stimulation treatment in medication-resistant major depression: a fast road to remission? J. Affect. Disord. 200, 6–14. 10.1016/j.jad.2016.04.015 27107779

[B18] Ebner N. C. Riediger M. Lindenberger U. (2010). FACES—A database of facial expressions in young, middle-aged, and older women and men: development and validation. Behav. Res. Methods 42, 351–362. 10.3758/BRM.42.1.351 20160315

[B19] Fonteneau C. Redoute J. Haesebaert F. Le Bars D. Costes N. Suaud-Chagny M. F. . (2018). Frontal transcranial direct current stimulation induces dopamine release in the ventral striatum in human. Cereb. Cortex 28, 2636–2646. 10.1093/cercor/bhy093 29688276 PMC5998959

[B20] George M. S. Ketter T. A. Post R. M. (1994). Prefrontal cortex dysfunction in clinical depression. Depression 2, 59–72. 10.1002/depr.3050020202

[B21] Gorwood P. (2008). Neurobiological mechanisms of anhedonia. Dialogues Clin. Neurosci. 10, 291–299. 10.31887/DCNS.2008.10.3/pgorwood 18979942 PMC3181880

[B22] Gould J. (1982). A psychometric investigation of the standard and short form beck depression inventory. Psychol. Rep. 51, 1167–1170. 10.2466/pr0.1982.51.3f.1167 7167614

[B23] Grimm S. Beck J. Schuepbach D. Hell D. Boesiger P. Bermpohl F. . (2008). Imbalance between left and right dorsolateral prefrontal cortex in major depression is linked to negative emotional judgment: an fMRI study in severe major depressive disorder. Biol. Psychiatry 63, 369–376. 10.1016/j.biopsych.2007.05.033 17888408

[B24] Hu Y. T. Hu X. W. Han J. F. Zhang J. F. Wang Y. Y. Wolff A. . (2021). Childhood trauma mediates repetitive transcranial magnetic stimulation efficacy in major depressive disorder. Eur. Arch. Psychiatry Clin. Neurosci. 271, 1255–1263. 10.1007/s00406-021-01279-3 34117915

[B25] Ikemoto S. (2007). Dopamine reward circuitry: two projection systems from the ventral midbrain to the nucleus accumbens–olfactory tubercle complex. Brain Res. Rev. 56, 27–78. 10.1016/j.brainresrev.2007.05.004 17574681 PMC2134972

[B26] Imbert L. Neige C. Moirand R. Piva G. Bediou B. Vallet W. . (2024). Eye-tracking evidence of a relationship between attentional bias for emotional faces and depression severity in patients with treatment-resistant depression. Sci. Rep. 14:12000. 10.1038/s41598-024-62251-4 38796509 PMC11127986

[B27] Isserles M. Shalev A. Y. Roth Y. Peri T. Kutz I. Zlotnick E. . (2013). Effectiveness of deep transcranial magnetic stimulation combined with a brief exposure procedure in post-traumatic stress disorder–a pilot study. Brain Stimul. 6, 377–383. 10.1016/j.brs.2012.07.008 22921765

[B28] Kato M. Okumura T. Tsubo Y. Honda J. Sugiyama M. Touhara K. . (2022). Spatiotemporal dynamics of odor representations in the human brain revealed by EEG decoding. PNAS 119:e2114966119. 10.1073/pnas.2114966119 35584113 PMC9173780

[B29] Kelly C. A. Freeman K. B. Schumacher J. A. (2022). Treatment-resistant depression with anhedonia: integrating clinical and preclinical approaches to investigate distinct phenotypes. Neurosci. Biobehav. Rev. 136:104578. 10.1016/j.neubiorev.2022.104578 35176319

[B30] Lefaucheur J. P. Aleman A. Baeken C. Benninger D. H. Brunelin J. Di Lazzaro V. . (2020). Evidence-based guidelines on the therapeutic use of repetitive transcranial magnetic stimulation (rTMS): an update (2014–2018). Clin. Neurophysiol. 131, 474–528. 10.1016/j.clinph.2019.11.002 31901449

[B31] Liu B. Zhang Y. Zhang L. Li L. (2014). Repetitive transcranial magnetic stimulation as an augmentative strategy for treatment-resistant depression, a meta-analysis of randomized, double-blind and sham-controlled study. BMC Psychiatry 14, 1–9. 10.1186/s12888-014-0342-4 25433539 PMC4264336

[B32] Marin R. S. Biedrzycki R. C. Firinciogullari S. (1991). Reliability and validity of the apathy evaluation scale. Psychiatry Res. 38, 143–162. 10.1016/0165-1781(91)90040-V 1754629

[B33] McIntyre R. S. Alsuwaidan M. Baune B. T. Berk M. Demyttenaere K. Goldberg J. F. . (2023). Treatment-resistant depression: definition, prevalence, detection, management, and investigational interventions. World Psychiatry 22, 394–412. 10.1002/wps.21120 37713549 PMC10503923

[B34] Midroit M. Chalençon L. Renier N. Milton A. Thevenet M. Sacquet J. . (2021). Neural processing of the reward value of pleasant odorants. Curr. Biol. 31, 1592–1605. 10.1016/j.cub.2021.01.066 33607032 PMC9291255

[B35] Mondino M. Poulet E. Brunelin J. (2021). Moving to accelerated protocols of tDCS in schizophrenia: a case report. Brain Stimul. 14, 822–824. 10.1016/j.brs.2021.05.006 34022429

[B36] Montgomery S. A. Åsberg M. (1979). A new depression scale designed to be sensitive to change. Br. J. Psychiatry 134, 382–389. 10.1192/bjp.134.4.382 444788

[B37] Mylius V. Ayache S. S. Ahdab R. Farhat W. H. Zouari H. G. Belke M. . (2013). Definition of DLPFC and M1 according to anatomical landmarks for navigated brain stimulation: inter-rater reliability, accuracy, and influence of gender and age. Neuroimage 78, 224–232. 10.1016/j.neuroimage.2013.03.061 23567888

[B38] Neige C. Imbert L. Beynel L. Fivel L. Mondino M. Brunelin J. (2024). Dual activation of the reward system using sensory-based intervention and non-invasive brain stimulation in depression: a way to move forward? Med. Hypotheses 189:111403. 10.1016/j.mehy.2024.111403

[B39] Neige C. Imbert L. Dumas M. Athanassi A. Thévenet M. Mandairon N. . (2023). Combining a breath-synchronized olfactometer with brain simulation to study the impact of odors on corticospinal excitability and effective connectivity. JoVE 203, 2–18. 10.3791/65714 38314795

[B40] O'Brien P. C. Fleming T. R. (1979). A multiple testing procedure for clinical trials. Biometrics 35, 549–556. 10.2307/2530245 497341

[B41] Peciña M. Sikora M. Avery E. T. Heffernan J. Peciña S. Mickey B. J. . (2017). Striatal dopamine D2/3 receptor-mediated neurotransmission in major depression: implications for anhedonia, anxiety and treatment response. Eur. Neuropsychopharmacol. 27, 977–986. 10.1016/j.euroneuro.2017.08.427 28870407 PMC5623119

[B42] Phillips R. D. Walsh E. C. Zürcher N. R. Lalush D. S. Kinard J. L. Tseng C. E. . (2023). Striatal dopamine in anhedonia: A simultaneous [11C]raclopride positron emission tomography and functional magnetic resonance imaging investigation. Psychiatry Res. Neuroimaging 333:111660. 10.1016/j.pscychresns.2023.111660 37301129 PMC10594643

[B43] Pogarell O. Koch W. Pöpperl G. Tatsch K. Jakob F. Mulert C. . (2007). Acute prefrontal rTMS increases striatal dopamine to a similar degree as D-amphetamine. Psychiatry Res. Neuroimaging 156, 251–255. 10.1016/j.pscychresns.2007.05.002 17993266

[B44] Pogarell O. Koch W. Pöpperl G. Tatsch K. Jakob F. Zwanzger P. . (2006). Striatal dopamine release after prefrontal repetitive transcranial magnetic stimulation in major depression: preliminary results of a dynamic [123I] IBZM SPECT study. J. Psychiatr. Res. 40, 307–314. 10.1016/j.jpsychires.2005.09.001 16259998

[B45] Rolls E. T. Kringelbach M. L. De Araujo I. E. (2003). Different representations of pleasant and unpleasant odours in the human brain. Eur. J. Neurosci. 18, 695–703. 10.1046/j.1460-9568.2003.02779.x 12911766

[B46] Rossini P. M. Barker A. T. Berardelli A. Caramia M. D. Caruso G. Cracco R. Q. . (1994). Non-invasive electrical and magnetic stimulation of the brain, spinal cord and roots: basic principles and procedures for routine clinical application. Report of an IFCN committee. Electroencephalogr. Clin. Neurophysiol. 91, 79–92. 10.1016/0013-4694(94)90029-9 7519144

[B47] Sabiniewicz A. Hoffmann L. Haehner A. Hummel T. (2022). Symptoms of depression change with olfactory function. Sci. Rep. 12:5656. 10.1038/s41598-022-09650-7 35383250 PMC8983665

[B48] Schulz K. F. Grimes D. A. (2005). Multiplicity in randomised trials II: subgroup and interim analyses. Lancet 365, 1657–1661. 10.1016/S0140-6736(05)66516-6 15885299

[B49] Sezille C. Fournel A. Rouby C. Rinck F. Bensafi M. (2014). Hedonic appreciation and verbal description of pleasant and unpleasant odors in untrained, trainee cooks, flavorists, and perfumers. Front. Psychol. 5:12. 10.3389/fpsyg.2014.00012 24478743 PMC3900918

[B50] Sezille C. Messaoudi B. Bertrand A. Joussain P. Thévenet M. Bensafi M. (2013). A portable experimental apparatus for human olfactory fMRI experiments. J. Neurosci. Methods 218, 29–38. 10.1016/j.jneumeth.2013.04.021 23660526

[B51] Silvanto J. Pascual-Leone A. (2008). State-dependency of transcranial magnetic stimulation. Brain Topogr. 21, 1–10. 10.1007/s10548-008-0067-0 18791818 PMC3049188

[B52] Snaith R. P. Hamilton M. Morley S. Humayan A. Hargreaves D. Trigwell P. (1995). A scale for the assessment of hedonic tone the Snaith–Hamilton Pleasure Scale. Br. J. Psychiatry 167, 99–103. 10.1192/bjp.167.1.99 7551619

[B53] Strafella A. P. Paus T. Barrett J. Dagher A. (2001). Repetitive transcranial magnetic stimulation of the human prefrontal cortex induces dopamine release in the caudate nucleus. J. Neurosci. 21:RC157. 10.1523/JNEUROSCI.21-15-j0003.2001 11459878 PMC6762641

[B54] Thomas-Danguin T. Rouby C. Sicard G. Vigouroux M. Farget V. Johanson A. . (2003). Development of the ETOC: a European test of olfactory capabilities. Rhinology 41, 134–151. Available at: https://www.rhinologyjournal.com/Rhinology_issues/400.pdf 14579654

[B55] Vedeniapin A. Cheng L. George M. S. (2010). Feasibility of simultaneous cognitive behavioral therapy and left prefrontal rTMS for treatment resistant depression. Brain Stimul. 3, 207–210. 10.1016/j.brs.2010.03.005 20965449 PMC2962866

[B56] Vida R. G. Sághy E. Bella R. Kovács S. Erdosi D. Józwiak-Hagymásy J. . (2023). Efficacy of repetitive transcranial magnetic stimulation (rTMS) adjunctive therapy for major depressive disorder (MDD) after two antidepressant treatment failures: meta-analysis of randomized sham-controlled trials. BMC Psychiat. 23:545. 10.1186/s12888-023-05033-y 37501135 PMC10375664

[B57] Wang Y. Cao N. Lin Y. Chen R. Zhang J. (2020). Hemispheric differences in functional interactions between the dorsal lateral prefrontal cortex and ipsilateral motor cortex. Front. Hum. Neurosci. 14:202. 10.3389/fnhum.2020.00202 32581747 PMC7283611

[B58] Wilkening J. Witteler F. Goya-Maldonado R. (2022). Suicidality and relief of depressive symptoms with intermittent theta burst stimulation in a sham-controlled randomized clinical trial. Acta Psychiatr. Scand. 146, 540–556. 10.1111/acps.13502 36163686

[B59] World Health Organization (2017). Depression and other common mental disorders: global health estimates (No. WHO/MSD/MER/2017.2). World Health Organization.

[B60] Young R. C. Biggs J. T. Ziegler V. E. Meyer D. A. (1978). A rating scale for mania: reliability, validity and sensitivity. Br. J. Psychiat. 133, 429–435. 10.1192/bjp.133.5.429 728692

[B61] Zimmerman M. Posternak M. A. Chelminski I. (2004). Defining remission on the Montgomery-Asberg depression rating scale. J. Clin. Psychiatry 65, 163–168. 10.4088/JCP.v65n0204 15003068

